# Relationship between caesarean section delivery and risk of overweight/obesity among children aged 6–23 months in the Tamale Metropolis of Ghana

**DOI:** 10.1017/jns.2022.39

**Published:** 2022-06-08

**Authors:** Issahaku Sulley, Mahama Saaka

**Affiliations:** School of Allied Health Sciences, University for Development Studies, P O Box TL 1883, Tamale, Ghana

**Keywords:** BMI/age *Z*-score, Caesarean section, Childhood overweight/obesity, Northern Ghana, ANC, antenatal care, AOR, adjusted odds ratio, BMI, body mass index, CI, confidence interval, COR, crude odds ratio, CS, caesarean section, CWC, Child Welfare Clinic, DBM, double burden of malnutrition, GIT, gastrointestinal tract, GWG, gestational weight gain, HAZ, height-for-age *Z*-score, WAZ, weight-for-age *Z*-score, WHZ, weight-for-height *Z*-score, WHO, World Health Organization

## Abstract

The recent exponential increase in caesarean section (CS) rates in many countries including Ghana requires an understanding of the potential long-term consequences on child health. The present study investigated the relationship between CS delivery and risk of childhood overweight/obesity. A retrospective cohort study was conducted from October 2019 to March 2020 in Ghana. Using multi-stage sampling, 553 mother–child pairs aged 6–23 months were selected from ten health facilities during child welfare clinic (CWC) services. We assessed the association between delivery mode (caesarean *v*. vaginal) and subsequent body mass index for age (BMI/age *Z*-score) using hierarchical multivariable linear regression analysis. The prevalence of overweight/obesity (BMI/age *Z*-score > +2 sd) in children was 3⋅6 %. After adjusting for maternal gestational weight gain, macrosomia and child feeding practices, children who were born through CS had mean BAZ which was 0⋅105 standard units significantly higher than their colleagues who were delivered through normal vaginal [beta coefficient (*β*) 0⋅105, (95 % CI 0⋅03, 0⋅55)]. CS birth was also associated with 3⋅2 times higher odds of overweight/obesity than vaginal delivery (AOR 3⋅23; 95 % CI 1⋅14, 9⋅13). Consequently, CS delivery was associated positively with increased body mass (adiposity) in the study sample. The association between CS delivery and risk of childhood obesity was attenuated after adjusting for macrosomia. These results would be important for informing clinicians and expectant mothers in considering CS delivery.

## Introduction

Over the past two decades, there has been an exponential increase in caesarean section (CS) deliveries in many countries including Ghana^(1)^. The prevalence of CS in the Northern region is 8⋅3 % as compared to 23⋅6 % in the Greater Accra region of Ghana^(2)^. CS is an important medical procedure for saving maternal and infant lives in emergency obstetric circumstances and has contributed to reducing maternal and neonatal mortality in low- and middle-income settings^(3)^. However, some health experts have questioned whether CS use beyond recommended levels has additional benefits.

CS delivery has been associated with early childhood overweight and obesity though this is not conclusive^(4–6)^. In 2016, the World Health Organization (WHO) estimated that 41 million children younger than 5 years were overweight or obese globally^(7)^. Two years later, estimates showed an increase to 38⋅3 million (5⋅6 %) overweight and obesity and 8⋅6 million experienced concurrent stunted growth and overweight^(8)^.

Overweight and obesity during childhood have been linked to adult morbidities such as cardiovascular disease, type 2 diabetes, orthopaedic problems, depression, low self-esteem and social marginalisation^(9–15)^.

Available evidence suggests that CS delivery disrupts the normal bacterial colonisation of the newborn^(16)^. In normal vaginal birth, the foetus is coated by, and exposed to bacterial strains from maternal vaginal and gastrointestinal tracts^(16,17)^. Children who are delivered by CS are less likely to have this exposure leading to lower transfer of maternal microbiota. This has been linked to poor development of child gut microbiota which is associated with weight gain in laboratory animals and may have similar manifestation in human children^(18)^.

Public healthcare providers need to understand the potential consequences of CS in order that appropriate interventions could be taken to address overweight/obesity-related morbidity and mortality. Unfortunately, limited studies have investigated the impact of CS delivery and childhood overweight/obesity in low- and middle-income countries. Therefore, the present study assessed the association between CS delivery and risk of childhood overweight/obesity among children 6–23 months in Northern Ghana.

## Methods

### Study setting

The present study was conducted in the Tamale Metropolis, one of the sixteen administrative districts in the Northern Region of Ghana. The overall land size of the Metropolis is estimated to be 646⋅90 km² with a total population of 233 252 consisting of 50⋅3 % females and 49⋅7 % males. Approximately 81 % of the people live in urban areas^(19)^. The Metropolis is divided into four Sub-Metropolitans, namely Bilpeila, Nyohini, Tamale Central and Vittin. Each sub-metro has a health management team supporting health facilities nearby.

### Study design, target population and sampling

A retrospective cohort study was used to collect the requisite data. The exposed group was mothers with CS delivery while the non-exposed group comprised of mothers with normal vaginal delivery. The primary respondents were mothers with children 6–23 months who were eligible for selection in both the exposed and control groups. The inclusion criteria were mother with children 6–23 months who sought postnatal care (PNC) services during routine monitoring and agreed to take part in the study. Children with an infection one week preceding the survey or those with known medical complications such birth asphyxia, and twin deliveries were also excluded.

The study used a multi-stage sampling procedure which involved first selecting a sub-district, health facilities and then mother–child pairs. Three of the four sub-districts were selected by a simple random sampling procedure. A complete list of both private and government health facilities in the selected sub-districts was compiled from which ten health facilities were carefully chosen at random. In each selected health facility, the comparison, as well as the exposed group members, was selected using a systematic random sampling procedure, where the PNC register served as the sampling frame. The first mother–child pair in each facility was selected by randomly choosing any number from 1 to the sampling interval. Subsequent mother–child pairs were selected by adding the sampling interval to the previously selected number in the sampling frame. This was done until the required sample size from each facility was obtained.

A minimum sample size of 482 (241 per study arm) was needed to have an 80 % power of detecting a significant difference of 8 % in the primary outcome measure between the study groups at 95 % confidence interval, and making provision of 10 % contingency, the sample size was adjusted to 530. However, 553 mother–child pairs were selected: 276 CS group and 277 vaginal delivery group.

### Data collection and tools

A structured pretested questionnaire was used for data collection through face-to-face interviews. Other vital information was extracted from the combined maternal and child health records booklet for both maternal and child health. The questionnaire was used to record data on demography background, economic status of household, antenatal care (ANC) or child welfare clinic (CWC) attendance, feeding practices of infant and young child among others.

The main tools used for data collection included an infantometer for measuring length/height and a Seca-uni electronic scale for measuring weight.

### Independent and dependent variables

The primary outcome measure was body mass index (BMI) for age *Z*-score (BAZ) which was also used to classify children into overweight/obesity (BMI/age *Z*-score > +2 sd). The main independent variable was mode of delivery (CS *v.* normal delivery) extracted from maternal antenatal clinical records.

### Potential confounding factors

Potential confounding variables measured in the study were socio-demographic characteristics including the age of child, mother parity, household wealth index, maternal BMI in early pregnancy, gestational age at delivery, maternal gestational weight gain during pregnancy, the occupational status, the mothers’ formal educational status, birth weight including the presence of macrosomia (defined as birth weight ≥4000 g) of the child, gender of child, the number of ANC visits before birth, infant feeding practices (e.g. breast-feeding, prelacteal feeding, colostrum feeding) which were assessed using 24-h dietary recall, childhood illnesses such as acute respiratory infection during 2 weeks preceding the survey.

The wealth index of households was measured based on housing quality and household assets as proxy indicators for socio-economic status (SES). The information collected on source of drinking water, housing quality (floor, walls and roof material), the presence of electricity, type of cooking fuel, livestock, type of toilet facility and ownership of modern household durable good (e.g. mobile phone, bicycle, sewing machine, television, car, radio, refrigerator, motorcycle, computer, mattress/bed, etc.) was used to calculate absolute household income status^(20)^. The amenities or durable goods are often considered as current goods that have been shown to reflect wealth status of households. The total scores of each respondent were classified as high and low household wealth index depending on the median cut-off point.

### Anthropometric measurements

Anthropometric measurements of length/height, weight and age were obtained following standardised techniques and equipment. The length of the infants (6–23 months) was measured with Seca infantometer to the nearest 0⋅1 cm in a recumbent (lying) position using a horizontal wooden length board and movable headpiece (infantometer). Child weight was measured using an electronic weighing scale (Seca 874) in only underwear or light clothing appropriate for the situation during measurements and recorded to the nearest 0⋅1 kg. The measurements were taken following WHO standard procedures and BAZ was calculated based on 2006 WHO growth charts^(21)^. The birth certificates and child health records booklets were used in determining child age which was usually recorded in months.

### Reliability and validity data

To ensure the reliability and validity of data, several procedures were used. All field enumerators and supervisors who had a minimum diploma qualification in a health-related subject received data collection training a week to the data collection in line with the objectives and methodology of the study. Pretesting of data collection tools including the weighing scale, infantometer and questionnaires were carried out. In all 5 % of the study, questionnaire were pretested and validated with mother–child pairs who were not part of the study. The field supervisors provided on-the-spot support to the enumerators. Field data collection supervisors checked questionnaires for completeness and consistency in the field and inconsistencies were rectified.

### Data analysis and presentation

Data were analysed using Statistical Package for Social Sciences (SPSS version 22). Means and standard deviations were used for continuous data while Chi-square test (*χ*^2^) was used for categorical data at the univariable level of analysis. In all analyses, a *P*-value of less than 0⋅05 was considered statistically significant. WHO Anthro software was used to convert measured weight, height and age of children into BMI for age *Z*-scores (BAZ). Before testing for associations between independent variables and the dependent outcomes, the data were cleaned, and outliers were removed. *Z*-scores which were outside the WHO flags: WHZ −5 to 5; HAZ −6 to 6 and WAZ −6 to 5 were also excluded from the dataset.

We performed several statistical analyses which provided complementary results. Analysis of covariance (ANCOVA) was used in examining the differences in the mean values of the quantitative outcome measures between the main explanatory variable while considering the influence of other covariates. Hierarchical multivariable linear regression analysis was used to test the relationship between delivery mode (caesarean *v*. vaginal) and our primary outcome (BMI/age *Z*-score). We also determined potential mediating effect of fetal macrosomia (birth weight ≥ 4⋅0 kg) on the relationship between CS delivery and childhood overweight/obesity by including it in the hierarchical multiple regression model in the second step while keeping other exposure variables in the first step of the model building. The percent of variability in the dependent variable that could be accounted for by all the predictors together was measured by R-square. The change in *R*^2^ is a way to evaluate how much predictive power was added to the model by the addition of another variable.

Adjusted odds ratios (AORs) and their associated 95 % confidence intervals (CIs) were also calculated using binary logistic regression analysis to assess the associations between caesarean birth and overweight/obesity. In the multivariable logistic regression analyses, only variables that showed evidence of association (*P* < 0⋅05) with the dependent variable in the univariate analysis were selected and adjusted for in the multivariable binary logistic regression analysis (Forward LR).

Covariates adjusted for were age of child, birth weight, gender, BMI of mother, maternal household wealth index, maternal education, maternal age, parity, gestational weight gain, gestational age at delivery, child feeding practices (e.g. breast-feeding status, introduction of solid foods, colostrum and prelacteal feeding).

Multicollinearity among variables included in the fully adjusted models was investigated using the variance inflation factor (VIF). VIF values greater than 5 were considered evidence of multicollinearity. Explanatory variables that were significant at bivariate analysis at a *P*-value of 0⋅05 or less were fed into the regression model after confirming the absence of multicollinearity between these independent variables.

### Ethical consideration and clearance

Ethical clearance for this study was obtained from the Institutional Review Board (IRB) of the Navrongo Health Research Centre with ethics approval identification number (NHRCIRB372). The ethics aspects of the study were also reviewed by the same scientific review committee that reviewed the study protocol. Permission was also obtained from the Tamale Metropolitan Health Directorate to conduct the study in the Sub-district health facilities. Written informed consent was obtained from literate participants who were also provided copies of the signed forms for their records and where the participants could not write or read, verbal informed consent was sought after providing the needed information and explanation.

## Results

### Socio-demographic characteristics of respondents

Table 1 presents the socio-demographic characteristics of the respondents in the study groups. The study groups were generally comparable, except for type of residence where mothers with CS deliveries tended to live in urban areas and households with high wealth index. More Christians than Moslems delivered by CS.

**Table 1.** Socio-demographic characteristics of the respondents (*N* 553)

Variables	Study groups		Test statistic
	Normal vaginal delivery	Caesarean section delivery	
Child sex	*n* (%)	*n* (%)	
Male	134 (50⋅2)	133 (49⋅8)	*χ*^2^ = 0⋅002 *P* = 0⋅97
Female	143 (50⋅0)	143 (50⋅0)	
Child age group (months)			
6–8	87 (51⋅8)	81 (48⋅2)	*χ*^2^ = 5⋅98 *P* = 0⋅05
9–11	68 (58⋅6)	48 (41⋅4)	
12–23	122 (45⋅4)	147 (54⋅6)	
Age of mother			
<25 years	59 (46⋅8)	67 (53⋅2)	*χ*^2^ = 0⋅94 *P* = 0⋅63
25–34 years	157 (51⋅8)	146 (48⋅2)	
≥35 years	61 (49⋅2)	63 (50⋅8)	
Marital status			
Currently not Married	10 (43⋅5)	13 (56⋅5)	*χ*^2^ = 0⋅42 *P* = 0⋅52
Currently Married	267 (50⋅4)	263 (49⋅6)	
Ethnicity			
Dagomba	212 (51⋅8)	197 (48⋅2)	*χ*^2^ = 5⋅66 *P* = 0⋅23
Mamprusi	22 (40⋅0)	33 (60⋅0)	
Gonja	26 (54⋅2)	22 (45⋅8)	
Akan	6 (31⋅6)	13 (68⋅4)	
Others	11 (50⋅0)	11 (50⋅0)	
Religion			
Muslim	242 (52⋅0)	223 (48⋅0)	*χ*^2^ = 4⋅46 *P* = 0⋅04
Christian	35 (39⋅8)	53 (60⋅2)	
Maternal education			
No formal education	144 (52⋅2)	132 (47⋅8)	*χ*^2^ = 2⋅43 *P* = 0⋅30
Low (Primary and JHS)	78 (51⋅3)	74 (48⋅7)	
High (At least SHS)	55 (44⋅0)	70 (56⋅0)	
Maternal Occupation			
Salary workers	28 (43⋅8)	36 (56⋅3)	*χ*^2^ = 1⋅25 *P* = 0⋅54
Non-salary workers	222 (51⋅2)	212 (48⋅8)	
Unemployed	27 (49⋅1)	28 (50⋅9)	
Type of residences			
Urban	85 (42⋅7)	114 (57⋅3)	*χ*^2^ = 9⋅48 *P* = 0⋅009
Rural	107 (58⋅5)	76 (41⋅5)	
Peri-Urban	85 (49⋅7)	86 (50⋅3)	
Household wealth index			
Low	81 (57⋅9)	59 (42⋅1)	*χ*^2^ = 4⋅52 *P* = 0⋅03
High	196 (47⋅5)	217 (52⋅5)	

### Past obstetric data and medical history of mothers

A greater proportion of CS delivery was observed among women who had adequate ANC attendance, compared with women whose ANC attendance was inadequate. Women who received at least three doses of SP also delivered through CS, compared with their colleagues who reported receiving less than three doses. CS delivery was prevalent among women who reported having *obstetric complications during pregnancy* (Table 2).

**Table 2.** Past obstetric data and medical history of mothers

Characteristic	Study groups		Test statistic
	Normal vaginal delivery	Caesarean section delivery	
	*n* (%)	*n* (%)	
Classification of Gravidity			
Primigravidae	57 (43⋅8)	73 (56⋅2)	Chi-squared (*χ*^2^) = 3⋅20 *P* = 0⋅20
Secundigravidae	60 (49⋅2)	62 (50⋅8)	
Multigravida	160 (53⋅2)	141 (46⋅8)	
Classification of Parity			
Primiparous	64 (46⋅4)	74 (53⋅6)	*χ*^2^ = 1⋅02 *P* = 0⋅60
Secundiparous	67 (51⋅5)	63 (48⋅5)	
Multiparous	146 (51⋅2)	139 (48⋅8)	
Gestational age at first ANC visit (months)			
First trimester (≤3 months)	192 (48⋅9)	201 (51⋅1)	*χ*^2^ = 0⋅8 *P* = 0⋅4
Beyond first trimester (>3 months)	85 (53⋅1)	75 (46⋅9)	
Gestation at delivery			
<37	45 (44⋅1)	57 (55⋅9)	*χ*^2^ = 1⋅79 *P* = 0⋅18
≥37	232 (51⋅4)	219 (48⋅6)	
Place of delivery			
Home delivery	48 (100⋅0)	0 (0⋅0)	Fisher's Exact Test = 52⋅37 *P* < 0⋅001
Institutional delivery	229 (45⋅3)	276 (54⋅7)	
Adequacy of prenatal care (at least 4 ANC visits and first ANC in first trimester of pregnancy)			
Inadequate	114 (58⋅2)	82 (41⋅8)	*χ*^2^ = 7⋅9 *P* = 0⋅005
Adequate	163 (45⋅7)	194 (54⋅3)	
TT injections			
No	34 (69⋅4)	15 (30⋅6)	*χ*^2^ = 8⋅01 *P* = 0⋅005
Yes	243 (48⋅2)	261 (51⋅8)	
SP Doses			
Less than 3	62 (56⋅4)	48 (43⋅6)	*χ*^2^ = 2⋅16 *P* = 0⋅14
At least 3	215 (48⋅5)	228 (51⋅5)	
Malaria during pregnancy			
No	232 (48⋅5)	246 (51⋅5)	*χ*^2^ = 3⋅41 *P* = 0⋅065
Yes	45 (60⋅0)	30 (40⋅0)	
Hb levels during first trimester			
Low (<11⋅0 g/dl)	106 (54⋅9)	87 (45⋅1)	*χ*^2^ = 11⋅03 *P* = 0⋅001
High (≥11⋅0 g/d)	65 (37⋅6)	108 (62⋅4)	
Obstetric complications during pregnancy?			
No	101 (80⋅2)	25 (19⋅8)	*χ*^2^ = 59⋅0 *P* < 0⋅000
Yes	176 (41⋅2)	251 (58⋅8)	

### Comparison of child and maternal anthropometric growth indicators with mode of delivery

Children born through CS delivery had higher birth weights than those born vaginally (3088⋅587 g *v*. 2864⋅719 g). However, CS delivery was associated negatively with mother's postpartum BMI and weight of mother in the first trimester (Table 3).

**Table 3.** Child and maternal anthropometric growth indicators according to mode of delivery

		*N*	Mean	Std. Deviation	95 % Confidence Interval for Mean		Test statistic
					Lower bound	Upper bound	
Body mass index for age	Normal vaginal delivery	277	−0⋅751	1⋅305	−0⋅91	−0⋅60	*F* (1, 551) = 18⋅31, *P* < 0⋅001
	Caesarean section	276	−0⋅270	1⋅339	−0⋅43	−0⋅11	
	Total	553	−0⋅511	1⋅343	−0⋅62	−0⋅40	
Current weight of mother (kg)	Normal vaginal delivery	277	61⋅32	10⋅10	60⋅13	62⋅52	*F* (1, 551) = 11⋅10, *P* = 0⋅001
	Caesarean section	276	58⋅58	9⋅24	57⋅49	59⋅68	
	Total	553	59⋅96	9⋅77	59⋅14	60⋅77	
Weight of mother on registration in the first trimester (kg)	Normal vaginal delivery	201	63⋅38	10⋅35	61⋅94	64⋅82	*F* (1, 416) = 9⋅78, *P* = 0⋅002
	Caesarean section	217	60⋅46	8⋅69	59⋅30	61⋅63	
	Total	418	61⋅87	9⋅63	60⋅94	62⋅79	
Birth weight of child (g)	Normal vaginal delivery	231	2864⋅719	535⋅379	2795⋅31	2934⋅12	*F* (1, 505) = 18⋅82, *P* < 0⋅001
	Caesarean section	276	3088⋅587	612⋅466	3016⋅01	3161⋅16	
	Total	507	2986⋅588	588⋅734	2935⋅22	3037⋅96	
Mother body mass index postpartum	Normal vaginal delivery	277	23⋅85	3⋅78	23⋅40	24⋅29	*F* (1, 551) = 10⋅27, *P* = 0⋅001
	Caesarean section	276	22⋅86	3⋅47	22⋅45	23⋅27	
	Total	553	23⋅35	3⋅66	23⋅05	23⋅66	
Height for age	Normal vaginal delivery	277	−0⋅790	1⋅795	−1⋅002	−0⋅58	*F* (1, 551) = 0⋅18, *P* = 0⋅68
	Caesarean section	276	−0⋅848	1⋅488	−1⋅03	−0⋅67	
	Total	553	−0⋅819	1⋅647	−0⋅96	−0⋅68	
Weight for height	Normal vaginal delivery	277	−0⋅800	1⋅247	−0⋅95	−0⋅65	*F* (1, 551) = 17⋅85, *P* < 0⋅001
	Caesarean section	276	−0⋅344	1⋅289	−0⋅50	−0⋅19	
	Total	553	−0⋅572	1⋅287	−0⋅68	−0⋅46	

### Determinants of BMI for age Z-score (multivariable regression analysis)

The unadjusted mean BMI for age *Z*-score (BAZ) of children born through CS was 0⋅48 more than children born through normal vaginal delivery (95 % CI 0⋅26, 0⋅70, *P* < 0⋅001). Using analysis of covariance (ANCOVA) that adjusted for colostrum feeding, breast-feeding status and maternal gestational weight gain during pregnancy BAZ remained significantly higher by 0⋅40 standard units (−0⋅278 *v*. −0⋅677) (95 % CI 0⋅14, 0⋅66, *P* = 0⋅002). However, there was a reduced difference in mean BAZ between CS and vaginal delivery after adjusting for macrosomia (−0⋅304 *v.* −0⋅591) (95 % CI−0⋅03, 0⋅55, *P* = 0⋅03).

Furthermore, in a hierarchical multivariable linear regression, we adjusted for confounders including birth weight, gender, age of child, current breast-feeding status, colostrum and prelacteal feeding. Children who were born through CS had mean BAZ which was 0⋅105 standard units significantly higher than their counterparts who were delivered through vaginal delivery [beta coefficient (*β*) 0⋅105 (95 % CI 0⋅03, 0⋅55)].

Current breast-feeding status was significantly and negatively associated with BMI/age *Z*-score [*β* (beta) −0⋅138 (95 % CI −3⋅64, −0⋅70)]. This means children who were breast-feeding were less likely to be overweight/obese. A unit increase in gestational weight gain led to decreased BAZ of −0⋅170 standard units [*β* −0⋅170 (95 % CI −0⋅10, −0⋅03)]. Children who were reported having been fed with colostrum had a higher mean BAZ (*β* 0⋅096, *P* = 0⋅045), compared to children who did not receive colostrum. Birth weight ≥4000 g (Macrosomia) was the strongest predictor of increased mean BAZ in children [*β* 0⋅208 (95 % CI 0⋅48, 1⋅29)].

In step 2 of the regression analysis, when macrosomia was added to the model, the percentage of variability accounted for went up from 8⋅2 to 12⋅4 % (*R*^2^ Change 0⋅042, *P* < 0⋅001) (Table 4).

**Table 4.** Risk factors of BMI for age *Z*-score

Model		Standardised coefficients	*t*	*P*-value	95⋅0 % Confidence Interval for *β*		Collinearity statistics	
		Beta			Lower bound	Upper bound	Tolerance	VIF
1	(Constant)		0⋅230	0⋅818	−1⋅89	2⋅39
	Caesarean section (CS) delivery	0⋅136	2⋅778	0⋅006	0⋅109	0⋅64	0⋅986	1⋅014
	Maternal gestational weight gain during pregnancy	−0⋅172	−3⋅535	<0⋅001	−0⋅10	−0⋅03	0⋅995	1⋅005
	Colostrum fed to child	0⋅099	2⋅036	0⋅042	0⋅05	3⋅06	0⋅990	1⋅010
	Currently breast-feeding	−0⋅152	−3⋅125	0⋅002	−3⋅88	−0⋅88	0⋅997	1⋅003
2	(Constant)		0⋅109	0⋅91	−1⋅98	2⋅21
	Caesarean section (CS) delivery	0⋅105	2⋅172	0⋅03	0⋅03	0⋅55	0⋅965	1⋅037
	Maternal gestational weight gain during pregnancy	−0⋅170	−3⋅577	<0⋅001	−0⋅10	−0⋅03	0⋅995	1⋅005
	Colostrum fed to child	0⋅096	2⋅009	0⋅045	0⋅03	2⋅97	0⋅990	1⋅010
	Currently breast-feeding	−0⋅138	−2⋅900	0⋅004	−3⋅64	−0⋅70	0⋅993	1⋅007
	Birth weight ≥4000 g (macrosomia)	0⋅208	4⋅310	<0⋅001	0⋅48	1⋅29	0⋅973	1⋅027

### Risk factors associated with childhood overweight/obesity in preschool-age children

Childhood overweight/obesity (BMI/age *Z*-score > +2 sd) was found in 3⋅6 % of the children aged 6–23 months and 9⋅3 % were macrosomic (≥4000 g) at birth. Table 5 shows the factors that associated with childhood overweight/obesity at the bivariate level. Prelacteal feeding, macrosomic birth weight, faulty start in complementary feeding and CS delivery associated positively with child's overweight/obesity status in preschool children.

**Table 5.** Risk factors associated with childhood overweight/obesity (bivariate analysis)

Factor	*N*	Classification of BMI for age		Test statistic
		Not overweight/obesity (≤2 sd)	Overweight/obesity (>2 sd)	
		*n* (%)	*n* (%)	
Mode of delivery				
Normal vaginal delivery	277	272 (98⋅2)	5 (1⋅8)	Chi-squared (*χ*^2^) = 5 2 *P* = 0⋅02
Caesarean section delivery	276	261 (94⋅6)	15 (5⋅4)	
Consumption of vitamin A-rich fruits and vegetables				
No	203	200 (98⋅5)	3 (1⋅5)	*χ*^2^ = 4⋅2 *P* < 0⋅04
Yes	350	333 (95⋅1)	17 (4⋅9)	
Timely initiation of breast-feeding				
No	198	187 (94⋅4)	11 (5⋅6)	*χ*^2^ = 3⋅3 *P* = 0⋅07
Yes	355	346 (97⋅5)	9 (2⋅5)	
Start complementary feeding at 6 months				
No	196	184 (93⋅9)	12 (6⋅1)	*χ*^2^ = 5⋅5 *P* = 0⋅02
Yes	357	349 (97⋅8)	8 (2⋅2)	
Child stunting				
Normal	425	415 (97⋅6)	10 (2⋅4)	*χ*^2^ = 8⋅4 *P* = 0⋅004
Stunted	128	118 (92⋅2)	10 (7⋅8)	
Birth weight				
Non-macrosomia	460	445 (96⋅7)	15 (3⋅3)	*χ*^2^ = 6⋅1 *P* = 0⋅01
Macrosomia	47	42 (89⋅4)	5 (10⋅6)	
Prelacteal feeding				
Yes	34	29 (85⋅3)	5 (14⋅7)	*χ*^2^ = 12⋅8 *P* < 0⋅001
No	519	504 (97⋅1)	15 (2⋅9)	

Before adjustment for potential confounders, CS delivery compared to vaginal delivery was associated with 3⋅13 (95 % CI 1⋅12, 8⋅73) times greater odds of overweight/obesity.

We then adjusted for potential confounding variables: maternal educational level, marital status, maternal age, ethnicity, infant sex, birth weight, gestational age, parity, early pregnancy BMI, mother's overweight in the third trimester, weight gain during pregnancy and child feeding practices.

The association between delivery mode and overweight/obesity in preschool children aged 6–23 months is shown in Table 6a. Childhood overweight/obesity was three times more common among the children born through CS (AOR 3⋅23; 95 % CI 1⋅14, 9⋅13, *P* = 0⋅027). Children with stunted growth were 3⋅6 times more likely to be overweight/obese compared to the normal children (AOR 3⋅62; 95 % CI 1⋅45, 9⋅04, *P* = 0⋅006). Overweight/obesity was higher among children who did not start complementary feeding at 6 months (AOR 2⋅81; 95 % CI 1⋅11, 7⋅10, *P* = 0⋅03) compared to children who were introduced to complementary foods at 6 months.

**Table 6.** Determinants of childhood overweight/obesity

	B	se	Wald	*P*-value	Adjusted odds ratio (AOR)	95 % CI for EXP(*β*)	
						Lower	Upper
(a)							
Caesarean section delivery	1⋅172	0⋅530	4⋅885	0⋅027	3⋅23	1⋅14	9⋅13
Faulty start of complementary feeding	1⋅034	0⋅473	4⋅788	0⋅029	2⋅81	1⋅11	7⋅10
Stunted growth	1⋅287	0⋅467	7⋅603	0⋅006	3⋅62	1⋅45	9⋅04
Constant	−4⋅960	0⋅591	70⋅507	<0⋅001	0⋅007		

	B	se	Wald	Sig.	Adjusted odds ratio (AOR)	95 % CI for EXP(B)	
						Lower	Upper
(b)							
Caesarean section delivery	0⋅824	0⋅542	2⋅312	0⋅128	2⋅28	0⋅79	6⋅59
Stunted growth	1⋅295	0⋅472	7⋅537	0⋅006	3⋅65	1⋅45	9⋅20
Faulty start of complementary feeding	1⋅115	0⋅482	5⋅360	0⋅021	3⋅05	1⋅19	7⋅84
Macrosomic children	1⋅226	0⋅577	4⋅519	0⋅034	3⋅41	1⋅10	10⋅56
Constant	−4⋅901	0⋅603	66⋅062	0⋅000	0⋅007		

Though we found an association between delivery mode and obesity, this association was attenuated (AOR 2⋅28; 95 % CI 0⋅79, 6⋅59, *P* = 0⋅13) when macrosomia was introduced into the regression model as a potential confounder. CS delivery thus became an insignificant predictor of childhood overweight/obesity (Table 6b).

## Discussion

The present study sought to determine the relationship between CS delivery and risk of childhood overweight/obesity among children 6–23 months in the Tamale Metropolis of Northern Ghana.

After adjusting for potential confounders, children who were born through CS had mean BAZ which was 0⋅105 standard units significantly higher than those who were born through normal vaginal delivery. Furthermore, childhood overweight/obesity was three times more common among the children born through CS. From both multivariable linear and logistic regression analyses, the effect of CS on childhood adiposity was reduced when macrosomia was adjusted for.

### Prevalence of childhood overweight/obesity

Childhood overweight/obesity (BMI/age *Z-*score > +2 sd) was found in 3⋅6 % of the children aged 6–23 months and 9⋅3 % were macrosomic (≥4000 g) at birth.

The prevalence of child overweight/obesity as measured in the present study was higher than the regional prevalence of 1⋅3 and 1⋅0 % in the Ghana demography and health survey and multiple indicator cluster survey, respectively^(22,23)^. The difference may be attributed to the differences in the study population characteristics. It is also important to note that the present study was conducted in predominately urban area of the region where food security and the use of milk substitute is likely to be higher than rural area where the previous two studies were conducted. The higher prevalence could also be explained by the rise in the level of CS delivery as reported in Ghana maternal and child health survey 2017^(2)^ and which is reported as a risk factor for overweight/obesity among preschool children in recent study undertaken in Poland^(24)^. Overall about 3 % of Ghanaian children under 5 years were reported overweight in Ghana demography and health survey 2014^(22)^ which is consistent with the finding of the present study. However, other previous studies reported a higher prevalence of overweight/obesity among preschool children in developing countries including Ghana as compared to the present study^(25)^, China^(26)^, Ethiopia^(27)^, Lebanon^(28)^, Iran^(29)^ and Vietnam^(30)^.

### Association between CS delivery and risk of childhood overweight/obesity

In the present study, we found a significant increased risk of childhood overweight/obesity among children delivered through CS as compared to those delivered vaginally. However, the positive association between CS delivery and risk of childhood obesity was attenuated when macrosomia was adjusted for. This suggests that macrosomia may correlate with CS delivery such that it reduced its effect. CS delivery may just be associated with large birth weight in this study population. While some studies including meta-analysis have consistently reported an association between caesarean delivery and childhood overweight/obesity, others found no association. Our finding is consistent with several previous studies conducted across the globe^(5,31–37)^. Of particular, relevance is a meta-analysis that summarised data from twenty-four studies and concluded that CS delivery is connected with higher risk of obesity compared to vaginal delivery^(38)^. The substantial risk of overweight or obesity associated with CS delivery buttresses the urgent need to rethink the practice.

However, some other studies have provided conflicting evidence on the association between non-elective CS delivery and early childhood overweight^(33,39)^. A study in Istanbul, Turkey showed no significant relationship between children delivered through CS and obesity^(39)^. The authors attributed the lack of association to the relatively higher formal education level, income and the confounding effect of household wealth that were not adequate considered^(39)^. Another possible factor could be the wider nature of the age group (2–14 years), a lot of factors may have contributed to the insignificant results found in the study. Another study conducted in Demark using Danish National Birth Cohort found lack of significant association between CS delivery and childhood overweigh/obesity but found antibiotic used during the first 6 month of life to be associated with childhood overweight/obesity^(36)^.

### Relationship between children's BMI for age and CS delivery

In the present study, CS delivery associated positively with mean BMI for age *Z*-score (BAZ) of children. However, there was a reduced significant difference in mean BAZ between CS and vaginal delivery when macrosomia was adjusted for in the regression model. The finding is consistent with results from other studies conducted in Brazil^(40)^, USA^(41)^, Demark^(6)^, Singapore^(33)^, Austria^(39)^ and Ireland^(36)^. The diminishing influence of CS delivery on the risk of obesity as child grow older has been attributed to the increasing influence of other risk factors of obesity like family dietary habit, physical inactivity and use of other electronic devices such as viewing television^(37)^.

### Proposed mechanisms explaining the observed association between CS delivery and subsequent obesity

A possible mechanism that has been hypothesised to explain the increasing risk of obesity and caesarean delivery could be related to environmental factors. Findings from some studies suggest that CS or vaginal may lead to difference in the initiation of laying foundation for oral and gut microbiota and consequently contribute to the health of the child^(16,42)^. During vaginal delivery, the baby comes in contact with mothers’ vagina which has vital flora for the infant's gastro intestinal track (GIT) colonisation or changes in the development and gut microbiota composition may affect host metabolism and energy storage and usage which can affect the development of obesity^(43)^. However, there is less direct contact between the mother and babies delivered through CS and so infants are less likely to derive the benefit of bacterial colonisation associated with normal vaginal delivery^(42)^. The changes in normal gut microbiota as a consequence of CS delivery are reported to be associated with weight gain in laboratory animals and may affect adiposity among CS-delivered children^(18)^.

Furthermore, studies have suggested that the initial flora in the GIT of infants could have influence in child breast-feeding. For instance, breast-feeding contributes to colonisation of infant gut and strongly influence the seeding of the gut especially CS-delivered children. Numerous studies have reported the late initiation of breast-feeding among CS children as compared to their counterparts with normal vaginal delivery^(44–47)^. There is also higher prelacteals feeding rate among CS-delivered babies^(48)^. Thus, the delay in initiation of breast-feeding, poor neonatal feeding and non-physiological start of GIT colonisation could contribute to childhood overweight/obesity.

### Other predictors of childhood overweight/obesity

Aside CS delivery, other factors were identified to be associated with childhood adiposity in the present study. Children who were breast-feeding had significantly lower mean BMI/age *Z*-score, compared to children who were not breast-feeding at the time of this study. This means children who were breast-feeding were less likely to be overweight/obese. Breast-feeding has been identified as a protective factor for childhood obesity in many studies including^(49–53)^, while other studies have provided conflicting evidence^(54,55)^. Early artificial feeding practices and a lower rate of breast-feeding initiation and shorter breast-feeding duration are consequences of caesarean delivery and may play a role in the biological pathway between caesarean delivery and later-life obesity.

Birth weight ≥4000 g (macrosomia) was the strongest predictor of mean BMI/age *Z*-score and macrosomic babies had significantly higher mean BMI/age *Z*-score as well as greater risk of childhood adiposity compared with babies with normal birth weight. These results are consistent with available evidence that suggests that fetal macrosomia is associated with increased risk of overweight and obesity in childhood and later-life stages^(56–59)^.

Gestational weight gain during pregnancy was negatively associated with BAZ. However, previous studies have reported positive associations between high gestational weight gain (GWG) and greater risk of childhood obesity and high BMI *Z*-scores especially among children aged at least 3 years^(60–62)^. The association between mode of delivery and adiposity may be confounded by maternal factors such as gestational weight gain that predisposes to CS and may therefore cause greater childhood body mass. The contrary finding in our sample may be due to low pre-pregnancy BMI of mothers in our sample or that the gestation weight gain was not excessive as reported in the other studies. In cases where a woman is underweight prior to pregnancy, any gestational weight may benefit the mother more than the growing foetus. Generally, obese women as a group gain less weight than non-obese women. Some studies have reported that GWG among obese and very obese women was lower than among non-obese women^(63,64)^ but it appears the GWG among obese women would be to the developing foetus. Overweight and obesity before pregnancy and an excessive GWG are associated with a greater risk of developing macrosomia.

Our analysis shows that, stunted children were 3⋅6 times more likely to be overweight/obese compared to the normal children. Previous studies showed similar results^(65–67)^. This association suggests the presence of double burden of malnutrition (DBM) in the study population. The relationship between stunting and overweight/obesity may be explained by the mechanism of growth retardation and changes in hormonal response. It has been hypothesised that stunted children have impaired fat oxidation, less lean body mass, resulting in decreased basal metabolic rate and physical activity relative to non-stunted children^(68)^. As a result, stunting can result in lower energy expenditure, more vulnerable to the effects of high fat intake, lower fat oxidation, disruption of food consumption adjustment and impaired fat metabolism^(69,70)^.

Overweight/obesity was also higher among children who did not start complementary feeding at the age of 6 months, compared to children who were introduced to complementary foods at 6 months. The association between mode of delivery and adiposity may be confounded by maternal and child factors including postpartum feeding behaviours (e.g. breast-feeding, complementary feeding). Introducing complementary feeding earlier may contribute to more rapid weight gain during infancy^(71–73)^ and increase the risk of childhood obesity in affluent populations^(74–77)^.

Since women with increased maternal pre-pregnancy, BMI are more likely to give birth to macrosomic babies^(78)^, such women are also prone to CS delivery. Undergoing CS delivery may predispose babies to artificial feeding which can also contribute to childhood obesity. At least one study has reported that increased maternal pre-pregnancy BMI and increased infant postnatal weight gain were independently associated with higher odds of early introduction of complementary feeding^(79)^.

## Limitations of the study

The interpretation of the findings of the present study should be made with some limitations in mind. The present study did not distinguish between elective and emergency (non-elective) caesarean delivery as the circumstances for each are different and may affect risk of childhood overweight differently. We also relied on the responses provided by the mothers over a long exposure period, and this may introduce recall bias which can affect the findings. Despite these limitations, our data have provided ample evidence on the relationship between CS delivery and overweight/obesity in Northern Ghana.

## Conclusion

The present study shows that CS delivery was associated with increased risks of high BMI-for-age and overweight/obesity among children 6–23 months in the Northern region of Ghana. The positive association between CS delivery and risk of childhood overweight/obesity was attenuated when macrosomia was adjusted for in the regression model. The results suggest the need for clinicians and patients to weigh the potential consequences when considering in particular the choice of elective CS delivery.
